# Reduced Interaction of Aggregated α-Synuclein and VAMP2 by Environmental Enrichment Alleviates Hyperactivity and Anxiety in a Model of Parkinson’s Disease

**DOI:** 10.3390/genes12030392

**Published:** 2021-03-10

**Authors:** Kyungri Kim, Soohyun Wi, Jung Hwa Seo, Soonil Pyo, Sung-Rae Cho

**Affiliations:** 1 Department and Research Institute of Rehabilitation Medicine, Yonsei University College of Medicine, Seoul 03722, Korea; kby930@yuhs.ac (K.K.); zugula@yuhs.ac (J.H.S.); neuro94@yuhs.ac (S.P.); 2 Brain Korea 21 PLUS Project for Medical Science, Yonsei University College of Medicine, Seoul 03722, Korea; 3 Department of Rehabilitation Medicine, Seoul National University College of Medicine, Seoul National University Hospital, Seoul 03080, Korea; wish118@yonsei.ac.kr; 4 Rehabilitation Institute of Neuromuscular Disease, Yonsei University College of Medicine, Seoul 03722, Korea; 5 Graduate Program of Nano Science and Technology, Yonsei University College of Medicine, Seoul 03722, Korea

**Keywords:** Parkinson’s disease, environmental enrichment, alpha-synuclein, vesicle-associated membrane protein 2, nucleus accumbens

## Abstract

Parkinson’s disease (PD) is a prevalent motor disease caused by the accumulation of mutated α-synuclein (α-Syn); however, its early stages are also characterized by non-motor symptoms, such as olfactory loss, cognitive decline, depression, and anxiety. The therapeutic effects of environmental enrichment (EE) on motor recovery have been reported, but its effects on non-motor symptoms remain unclear. Herein, we reveal the beneficial effects of EE on PD-related non-motor symptoms and changes in synaptic plasticity in the nucleus accumbens. To investigate its therapeutic effects in the early phase of PD, we randomly assigned eight-month-old mice overexpressing human A53T (hA53T) α-Syn to either the EE or standard condition groups for two months. Next, we performed behavioral tests and biochemical and histological analyses at 10 months of age. EE significantly alleviated locomotor hyperactivity and anxiety during the early stages of PD. It normalized the levels of tyrosine hydroxylase, phosphorylated and oligomeric α-Syn, and soluble *N*-ethylmaleimide-sensitive factor attachment protein receptor complex-forming proteins, including synaptosomal-associated protein, 25 kDa, syntaxin1, and vesicle-associated membrane protein 2 (VAMP2). Moreover, the interactions between VAMP2 and pSer129 α-Syn were markedly reduced following EE. The restoration of synaptic vesicle transportation status may underlie the neuroprotective effects of EE in hA53T α-Syn mice.

## 1. Introduction

Alpha-synuclein (α-Syn) is the main constituent of the neuropathological lesions found in patients with Parkinson’s disease (PD), Lewy body dementia, multiple system atrophy, and other disorders collectively known as α-synucleinopathies [1,2,3]. Alpha-synuclein (α-Syn) is a protein that is widely distributed in the presynaptic nerve terminal, and its role has not yet been fully elucidated. However, there is accumulating evidence regarding its relationship with vesicle trafficking. The number of presynaptic vesicles decreases when α-Syn levels decrease in primary hippocampal neurons [4]. Moreover, aggregated α-Syn acts as a negative regulator of neurotransmitter release in mice expressing mutant human α-Syn [5,6]. Although normal levels of α-Syn assist with vesicle trafficking, α-Syn overexpression interferes with vesicle trafficking [7] and neurotransmitter release by inhibiting the recycling of synaptic vesicle proteins [8,9]. α-Syn directly binds to synaptobrevin-2, vesicle-associated membrane protein 2 (VAMP2) [10], and acidic lipid-containing membranes [11].

In particular, the A53T mutation of α-Syn is a point mutation that causes familial PD. There are more Lewy bodies and higher levels of phosphorylated α-Syn in human A53T (hA53T) mutant mice [4,5]. Mice with PD overexpressing hA53T α-Syn show progressive motor [12,13] and non-motor dysfunctions [14,15,16]. Non-motor symptoms, such as hyposmia [14], hyperactivity [15], and anxiety [14,16], have been found in 7–12-month-old transgenic mice. Dysfunction of axonal transportation by synaptophysin-positive vesicles has also been shown in the hA53T transgenic α-Syn model [17].

The pathogenesis of PD is associated with the dysfunction of various brain regions, such as the substantia nigra pars compacta (SNpc), striatum, and cortex, with an increased degree of α-Syn pathology [18]. Among these brain regions, the nucleus accumbens (NAc), a part of the ventral striatum, is the brain region that is mainly responsible for reward and emotional processes and it has been implicated in psychiatric and neurodegenerative diseases, such as PD [19,20,21]. The manifestation of non-motor symptoms is related to neurochemical changes and the brain reward circuit, including the NAc in PD [19,22]. Unger et al. confirmed the increase in locomotor hyperactivity in mice with an A53T mutation and the decrease in dopamine transporter in the NAc [15]. Given the increasing interest in the NAc, various treatments and interventions specific to this brain region have been conducted to alleviate the non-motor symptoms of PD [23,24,25].

We utilized environmental enrichment (EE) as a potential treatment for PD in this study. EE is a method of breeding animals in a large cage containing running wheels and novel objects, and providing social interactions in the form of a complex stimulus mixture comprising physical, cognitive, and social experiences [26]. In clinical studies, EE has been used as rehabilitation therapy for patients [27,28]. Epidemiological studies have supported a link between hard exercise and a reduced risk of PD [29,30]. Additionally, many studies examining the effects of exercise on normal aging or PD support the advantages of exercise, physical activity, and EE [31,32,33]. The therapeutic effects of EE in the behavioral recuperation of motor function in a mouse model of PD pathology induced by the administration of 1-methyl-4-phenyl-1,2,3,6-tetrahydrophyridine (MPTP) have been reported [34,35]. However, there have been no studies, to our knowledge, regarding the effects of EE on non-motor symptoms during the pre-motor phase in mice overexpressing hA53T α-Syn. Through a transgenic mouse model of PD, this study showed the influence of EE on the induction of synaptic plasticity in the striatum and NAc in the initial phase of PD.

## 2. Materials and Methods

### 2.1. Transgenic Mouse Model of Parkinson’s Disease

The hA53T α-Syn transgenic line G2-3 mice (B6.Cg-Tg (Prnp-SNCA*A53T) 23Mkle/J; Jackson Laboratories, stock no. 006823, Bar Harbor, ME, USA) were bred with wild-type (WT) mice to obtain WT and hemizygous mice. All animals were raised in a facility qualified by the Association for Assessment and Accreditation of Laboratory Animal Care (AAALAC) and provided with food and water ad libitum with a 12-h light/dark cycle (8:00–20:00 light, 20:00–8:00 dark) according to the regulations for animal protection. All the behavioral experiments were conducted during the light cycle. The experimental procedures were approved by the Yonsei University Health System Institutional Animal Care and Use Committee (YUHS-IACUC approval number 2017-0039). 

### 2.2. Genotyping

Genotyping of mice was performed according to the manufacturer’s protocol (Jackson Laboratories). Genomic DNA was obtained from a 2 mm piece of each three-week-old mouse’s tail using a prepGEM Tissue Kit (ZyGEM, PUN0100, Hamilton, New Zealand). The mouse tail tissue was incubated with 1 μL of prepGEM, 10 μL of Buffer Gold, and 89 μL of autoclaved deionized distilled water at 75 °C for 15 min and 95 °C for 5 min. The following primers were used for polymerase chain reaction (PCR): forward, 5′-TCA TGA AAG GAC TTT CAA AGG C-3′; transgene reverse, 5′-CCT CCC CCA GCC TAG ACC-3′ (transgene = ~500 bp); internal positive control forward, 5′-CTA GGC CAC AGA ATT GAA AGA TCT-3′; and internal positive control reverse, 5′-GTA GGT GGA AAT TCT AGC ATC C-3′ (internal positive control = 324 bp). Electrophoresis was performed by loading 10 μL of each PCR product onto a 1.5% agarose gel.

### 2.3. Housing Conditions 

At eight months of age, the mice were randomly separated into standard cages (SC) or subjected to EE for two months. Standard housing environments included common housing cages (27 × 22.5 × 14 cm^3^) without any novel objects. The control mice were housed for two months in SC (five mice/cage). The mice subjected to EE were housed in a large cage (86 × 76 × 31 cm^3^) that included tunnels, shelters, toys, and running wheels for voluntary exercise, and conditions allowing for social interactions (10 mice/cage) for the same duration. The subjects were relocated once per week.

### 2.4. Behavioral Assessment

All behavioral tests were conducted on mice aged eight and 10 months.

#### 2.4.1. Grip Strength Test 

A grip strength test was performed using the SDI Grip Strength System (San Diego Instruments Inc., San Diego, CA, USA), which consists of a push-pull strain gauge. Each animal grasped a triangular metal wire (2 mm in diameter) with its forepaws and its tail was pulled until the animal lost its grip on the wire. The machine automatically recorded the peak force in gram-force.

#### 2.4.2. Hanging Wire Test 

The mice were hung by their forepaws from a horizontal rod (5 × 5 mm^2^ area, 35 cm length, between two poles 50 cm high). The mice tended to use their hind limbs to prevent themselves from falling and to aid their progression along the wire. For this test, the latencies to fall from the wire were recorded for 60 s [36].

#### 2.4.3. Cylinder Test 

When mice are placed in a transplant plexiglass cylinder 8 cm in diameter and 18 cm in height, they spontaneously stood up and use their forepaws for support. For this test, the number of forelimbs touching the cylinder wall (Jeung Do B&P, Seoul, Korea) while the animal stood up was counted over a period of 5 min [37].

#### 2.4.4. Open Field Test

An open field test was performed for 25 min when the mice were 10 months old to determine whether EE exposure influenced locomotor activity. Activity was recorded in a square area measuring 30 × 30 × 30 cm^3^. The total distance traveled by the mice was recorded for 25 min. The floor of the area was composed of 16 sectors. The four inner sectors represented the center and the 12 outer sectors, the periphery. The total time spent in the 12 outer sections was recorded as a sign of anxiety [38,39]. Mice were individually placed in the periphery of the area and allowed to explore spontaneously for 25 min while being recorded with a video camera. The resulting data were obtained and analyzed using the Smart Vision 2.5.21 (Panlab, Barcelona, Spain) video tracking system.

### 2.5. RNA Extraction

Mice were euthanized and perfused with cold 1 × phosphate buffered saline at 10 months of age. Total RNA was extracted from the striatum and NAc of the mouse brains using TRIzol reagent (Invitrogen Life Technologies, Carlsbad, CA, USA). The purity of the extracted RNA was assessed using a Nanodrop-2000 spectrophotometer (Thermo Fisher Scientific, Waltham, MA, USA). Purified total RNA (1 μg) was used as a template to produce complementary DNA (cDNA) using the ReverTra Ace qPCR RT master mix with gDNA remover (TOYOBO, FSQ-301, Osaka, Japan).

### 2.6. Quantitative Real Time Polymerase Chain Reaction (qRT-PCR)

The PCR reactions were performed using 1 μL of cDNA in a total volume of 20 μL. qRT-PCR was performed in triplicate on a LightCycler 480 (Roche Applied Science, Mannheim, Germany) using the LightCycler 480 SYBR Green master mix (Roche Applied Science, PB20.12–20, Mannheim, Germany) with the following cycling conditions: amplifications were started with a 5-min template preincubation step at 95 °C, followed by 40 cycles at 95 °C for 20 s, 62 °C for 20 s, and 72 °C for 15 s. Melting curve analysis was initiated at 95 °C for 5 s, followed by 1 min at 60 °C. The specificity of the product was confirmed by melting curve analysis, which showed a distinctive single sharp peak with the expected melting temperature for all samples. The glyceraldehyde-3-phosphate dehydrogenase (GAPDH) gene was used as an internal control. The relative expression level of each gene of interest was determined using the 2^−ΔΔCt^ method. 

### 2.7. Western Blot Analysis 

The striatum and NAc were lysed in 500 μL of cold RIPA buffer (50 mM Tris-HCl, pH 7.5, 1% Triton X-100, 150 mM NaCl, 0.1% sodium dodecyl sulfate (SDS), and 1% sodium deoxycholate) with a protease inhibitor cocktail (Sigma Aldrich, 5871S, St. Louis, MO, USA). The tissue lysates were centrifuged at 16,000× *g* at 4 °C for 20 min; the supernatants were extracted and the protein contents were analyzed using the Bradford method. Extracted proteins (50 μg) were dissolved in sample buffer (Invitrogen Life Technologies, 500-0205, Carlsbad, CA, USA), incubated for 10 min at 80 °C, and separated on a 4–12% SDS reducing polyacrylamide gel (Invitrogen Life Technologies, NP0322, Carlsbad, CA, USA). The separated proteins were equally loaded and transferred onto polyvinylidene difluoride membranes (Invitrogen Life Technologies, LC2002, Carlsbad, CA, USA) using a trans-blot system (NovexR Mini-Cell; Invitrogen Life Technologies, 110501-1021, Carlsbad, CA, USA). The blots were blocked for 1 h in Tris-buffered saline (TBS) (10 mM Tris-HCl, pH 7.5, 150 mM NaCl) containing 5% nonfat dry milk (Bio-Rad, 232100, Berkeley, CA, USA) at 25 °C and washed three times with TBS. Then, the blots were incubated at 4 °C overnight with the following antibodies: antibodies against synaptosomal-associated protein, 25 kDa (SNAP-25, 1:1000, Abcam, ab5666, Cambridge, UK); syntaxin1 (1:1000, Santa Cruz Biotechnology, Santa Cruz, sc-12736, CA, USA); VAMP2 (1:1000, Abcam, ab181869, Cambridge, UK); solute carrier family 6 (neurotransmitter transporter, dopamine), member 3 (Slc6a3, 1:1000, Chemicon, AMT-003, Pemecula, CA, USA); dopamine receptor D1 (Drd1, 1:1000, Proteintech, 17934-1-AP, Manchester, UK); Drd2 (1:1000, Abcam, ab32349, Cambridge, UK); α-Syn (1:1000, Abcam, ab138501, Cambridge, UK); human pSer129 α-Syn (1:1000, Abcam, ab168381, Cambridge, UK); and tyrosine hydroxylase (TH, 1:1000, Sigma Aldrich, T1299, St. Louis, MO, USA) diluted in Tris-buffered Saline, 0.1% TWEEN® 20 (10 mM Tris pH 7.5, 150 mM NaCl, and 0.02% Tween 20) with 3% nonfat dry milk. After incubation, the blots were rinsed three times with TBST and incubated for 1 h with horseradish peroxidase-conjugated secondary antibodies (1:3000, Santa Cruz Biotechnology, anti-rabbit; sc-2357, anti-mouse; sc-516102, Santa Cruz, CA, USA) at room temperature. Antibodies against the housekeeping gene (actin) were also used (1:1000, Santa Cruz Biotechnology, sc-47778, Santa Cruz, CA, USA). After washing with TBST, the blots were visualized using an enhanced chemiluminescence (ECL) detection system (Sigma Aldrich, RPN2109, St. Louis, MO, USA). The Western blot results were analyzed using the Multi Gauge system (Fuji Photo Firm, version 3.0, Tokyo, Japan).

### 2.8. Immunohistochemistry (IHC)

The animals were euthanized and perfused with 4% paraformaldehyde (PFA) in 0.1 M phosphate buffer, pH 7.4. Their brains were removed and post-fixed for 1 h, followed by cryoprotection in 30% sucrose in TBS containing 0.02% sodium azide. The harvested brain tissues were cryo-sectioned at a thickness of 16 μm along the sagittal or coronal plane, and IHC analysis was performed on four sections. For immunofluorescence double labeling, the sections were stained with the following antibodies: antibodies against human α-Syn (1:400, Abcam, ab138501, Cambridge, UK); human pSer129 α-Syn (1:100, Abcam, ab168381, Cambridge, UK); and Slc6a3 (1:400, Chemicon, AMT-003, Pemecula, CA, USA), and secondary antibodies, such as Alexa Fluor® 488 goat anti-rabbit (1:400, Invitrogen, A110088, Carlsbad, CA, USA), Alexa Fluor® 594 anti-mouse (1:400, Invitrogen, A11005, Carlsbad, CA, USA), and Alexa Fluor® 594 anti-rat (1:400, Abcam, ab150160, Cambridge, UK). The sections were mounted on glass slides with fluorescent mounting medium containing 4′,6-diamidino-2-phenylindole (Vector, H-1200; Vector, Burlingame, CA, USA). To examine the expression of tyrosine hydroxylase (TH), experiments were performed as described in previous studies [40,41]. The sections were then permeabilized with 1% bovine serum albumin and 0.5% Triton-X in phosphate buffered saline for 30 min. After overnight incubation with anti-TH antibodies (1:2000, Sigma Aldrich, T1299, St. Louis, MO, USA), the sections were washed with 0.1 M phosphate buffer solution and biotinylated with alkaline phosphatase-conjugated secondary antibody (1:400, Vectorshield, Vector, BA-100, Burlingame, CA, USA) for 1 h, followed by treatment with peroxidase conjugated with avidin-biotin complexes in phosphate buffer solution (1:200; Vectorshield, Vector, PK-6100, Burlingame, CA, USA). The immunostaining was visualized using 0.01% 3,3-diaminobenzidine (Sigma Aldrich, D5637-1G, St. Louis, MO, USA) and 0.012% hydrogen peroxide in phosphate buffer solution. The stained sections were examined using a fluorescence microscope (Axio Imager M2, Zeiss, Gottingen, Germany) and a confocal microscope (LSM700, Zeiss, Gottingen, Germany).

### 2.9. In Situ Proximity Ligation Assay (PLA)

Animals were euthanized and perfused with 4% PFA in 0.1 M phosphate buffer, pH 7.4. Their brains were removed and post-fixed for 1 h, followed by cryoprotection in 30% sucrose in TBS containing 0.02% sodium azide. The harvested brain tissues were cryo-sectioned at a thickness of 16 μm along the sagittal or coronal plane. The sections were stained with the following primary antibodies: antibodies against VAMP2 (1:400, Abcam, ab181869, Cambridge, UK) and human pSer129 α-Syn (1:1000; FUJIFILM Wako Pure Chemical Corporation, 015-25191, Tokyo, Japan), overnight at 4 °C to detect the interactions between the VAMP2 and human pSer129 α-Syn proteins. After rinsing, the sections were simmered with the secondary oligonucleotide-linked antibodies (The Duolink® kit, DUO92102, Olink Bioscience, Uppsala, Sweden) provided in the kit. The oligonucleotides attached to the antibodies were detected using a fluorescent probe (Detection Kit 563). The specks were detected using confocal imaging (LSM700, Zeiss, Oberkochen, Germany). The samples were analyzed and figures prepared using ZEN software (ZEN 3.0 blue edition, Zeiss, Oberkochen, Germany).

### 2.10. Statistical Analysis 

Statistical analysis was conducted using the Statistical Package for Social Sciences (SPSS) software (IBM Corp., released in 2017) and IBM SPSS Statistics for Windows (version 25.0. software, IBM Corp., Armonk, NY, USA). Data are expressed as the mean ± standard error of the mean. The results of behavioral tests, qRT-PCR, Western blotting, and IHC were analyzed by one-way analysis of variance followed by a post-hoc Bonferroni test to adjust the variance for multiple testing effects (WT control group (WT-CON), WT-EE, PD-SC, and PD-EE). Statistical significance was set at *p* < 0.05.

## 3. Results

### 3.1. EE Ameliorates Hyperactivity and Anxiety but Not Motor Function in hA53T α-Syn-Overexpressing Transgenic Mice

**Figure 1:**
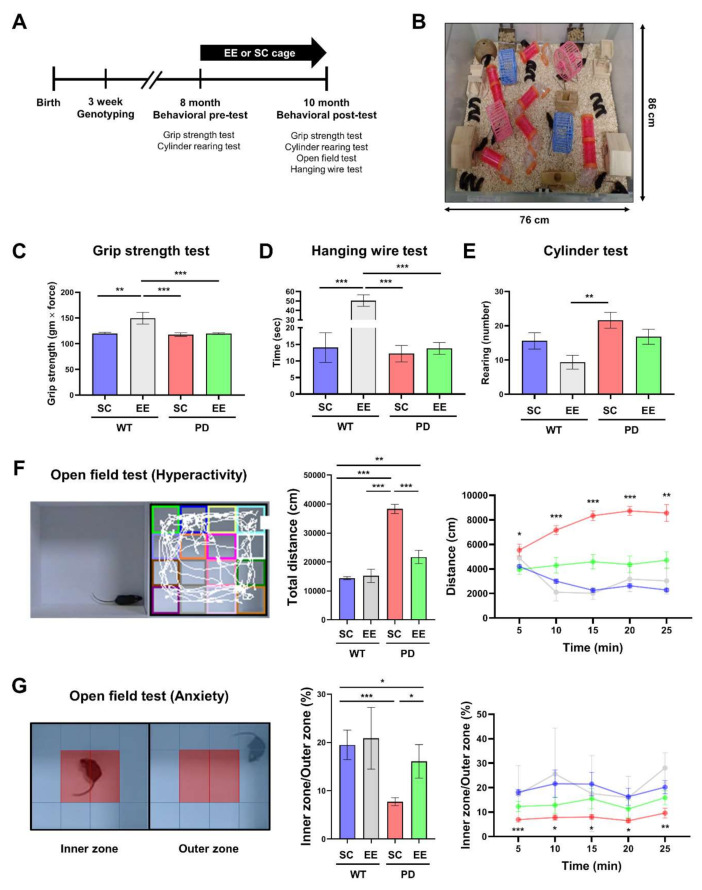
Environmental enrichment (EE) ameliorates hyperactivity and anxiety but not motor function in human A53T(hA53T) alpha-synuclein (α-Syn) mice. (**A**) Experimental scheme. (**B**) EE (86 × 76 × 31 cm^3^). (**C**) The grip strength test (wild-type (WT)- standard cages (SC), *n* = 5; WT-EE, *n* = 10; Parkinson’s disease (PD)-SC, *n* = 10; PD-EE, *n* = 10). (**D**) The hanging wire test (WT-SC, *n* = 5; WT-EE, *n* = 5; PD-SC, *n* = 10; PD-EE, *n* = 10). (**E**) The cylinder test (WT-SC, *n* = 10; WT-EE, *n* = 8; PD-SC, *n* = 10; PD-EE, *n* = 16). (**F**, **G**) Open field test conducted in a square area (30 × 30 × 30 cm^3^). The floor was divided into 16 sectors. The four red sectors represent the inner zone, and the 12 blue sectors represent the outer zone. (**F**) Open field test for hyperactivity (WT-SC, *n* = 12; WT-EE, *n* = 3; PD-SC, *n* = 15; PD-EE, *n* = 12). (**G**) Open field test for anxiety (WT-SC, *n* = 10; WT-EE, *n* = 3; PD-SC, *n* = 12; PD-EE, *n* = 11. The values represent the mean ± SEM. * *p* < 0.05, ** *p* < 0.01, and *** *p* < 0.001.

Eight-month-old WT or hA53T α-Syn mice were randomly allocated to either the EE group (WT-EE, PD-EE) or SC group (WT-SC, PD-SC) (*n* = 15–25 per group) for two months (Figure 1A,B). Motor function tests were performed to determine whether motor symptoms appeared in hA53T α-Syn mice at 10 months of age. The grip strength results showed no significant differences among groups except for the WT-EE group. There were significant differences between the WT-EE group and the other groups (Figure 1C). The endurance time in the hanging wire test was not significantly different between all groups except for the WT-EE group, which showed significant differences compared to those in the other groups (Figure 1D). There were no significant differences in the grip strength and hanging wire tests between the PD-SC and PD-EE groups, whereas there were significant differences in those between the WT-SC and WT-EE groups. The rearing count in the cylinder test was only different between the WT-EE and PD-SC groups (Figure 1E).

The open field test is commonly used to estimate locomotor activity and spontaneous exploration in a new environment [42,43]. The total distance traveled by each mouse was recorded for 25 min as an index of hyperactivity [44]. During the 25 min, the total distance traveled significantly increased in the PD-SC group compared to the other groups (Figure 1F). This indicates that long-term EE exposure can ameliorate PD-associated hyperactivity. To determine whether EE exposure affected anxiety, the proportion of time spent in the inner zone compared to the outer zone was examined (Figure 1G). The proportion of time spent in the inner zone was significantly lower in the PD-SC group than in the WT-SC and PD-EE groups. This demonstrates that long-term EE exposure reduces anxiety in PD.

### 3.2. EE Reduces the Degeneration of Dopaminergic Nerve Terminals in the NAc of hA53T α-Syn-Overexpressing Transgenic Mice

**Figure 2:**
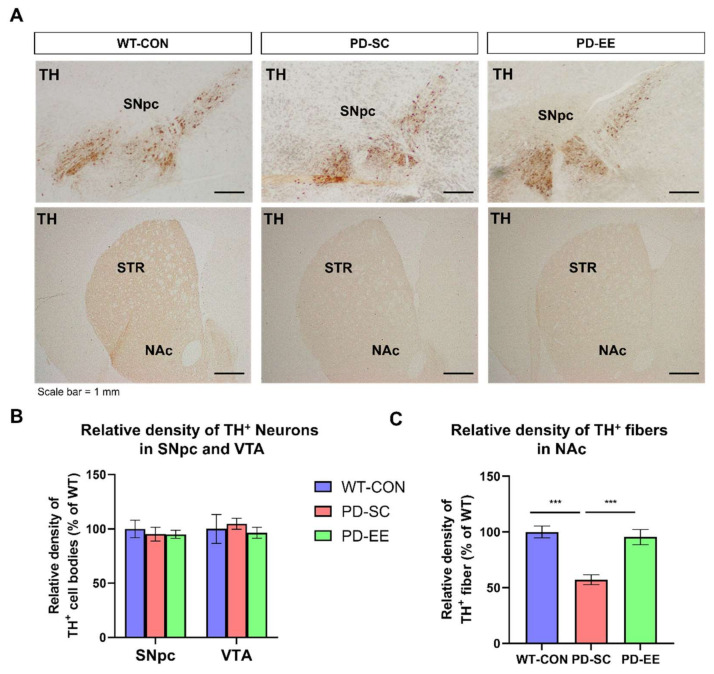
EE prevents the degeneration of dopaminergic nerve terminals in the nucleus accumbens (NAc) of hA53T alpha-synuclein (α-Syn) mice. (**A**) Representative images of tyrosine hydroxylase (TH)-positive cells in substantia nigra pars compacta (SNpc) and ventral tegmental area (VTA) in the three groups (scale bar = 1 mm). (**B**) Relative density of TH-positive cells in the SNpc and VTA (SNpc: WT control group (WT-CON), *n* = 8; PD-SC, *n* = 7; PD-EE, *n* = 3 and VTA: WT- CON, *n* = 5; PD-SC, *n* = 3; PD-EE, *n* = 3). (**C**) Relative density of TH-positive dopaminergic nerve terminals in the NAc (*n* = 5, each). *** *p* < 0.001 versus WT- CON; one-way ANOVA followed by post-hoc comparison. Data in all panels represent the mean ± SEM.

To investigate whether dopaminergic neurons in the SNpc and the ventral tegmental area (VTA) were degenerated at 10 months of age, immunostaining was performed to analyze TH-positive cells (Figure 2A). The immunostaining results showed that TH-positive cell bodies (% of WT control group) in SNpc did not change significantly among the three groups (WT control group, PD-SC, and PD-EE). Additionally, TH-positive cell bodies (% of WT control group) in the VTA did not differ significantly between the three groups (Figure 2B).

Immunostaining was performed to analyze TH-positive cell density to investigate whether dopaminergic nerve terminals in the NAc were degenerated at 10 months of age and examine the effects of EE exposure on dopaminergic nerve terminals. Immunostaining results showed that the number of dopaminergic nerve terminals in the striatum decreased significantly in mice from the PD-SC and PD-EE groups, compared to that in the mice from the WT control group (Figure 2C).

### 3.3. EE Increases Soluble N-Ethylmaleimide-Sensitive Factor Attachment Protein Receptor (SNARE) Expression and Alters Dopamine Transporters and Receptors in the NAc of hA53T α-Syn-Overexpressing Transgenic Mice

**Figure 3:**
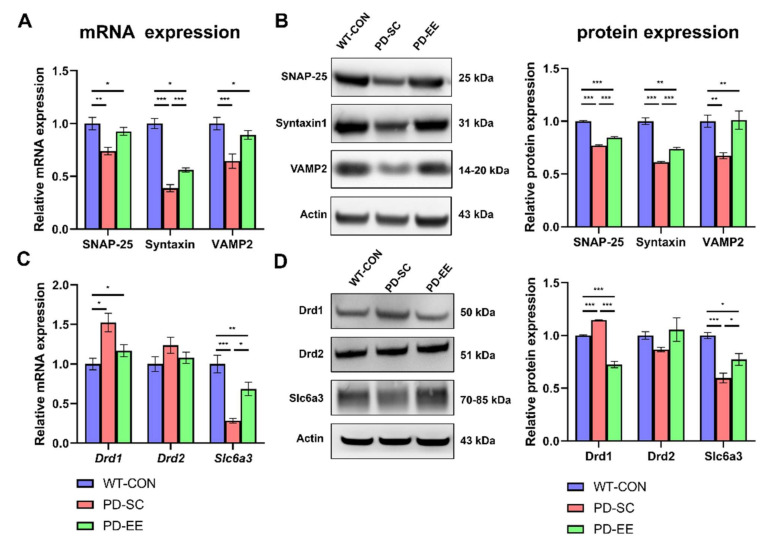
EE restores soluble *N*-ethylmaleimide-sensitive factor attachment protein receptor (SNARE) expression and alters the expression of Slc6a3 and dopamine receptors in the NAc of hA53T α-Syn-overexpressing transgenic mice. (**A**) qRT-PCR results showed that the relative mRNA expression of SNAP-25, syntaxin1, and VAMP2 was significantly increased in mice from the PD-EE group, compared to that in mice from the PD-SC group (*n* = 4, each). (**B**) Western blot results showed the relative protein expression levels of both syntaxin1 and VAMP2 to be significantly increased in mice from the PD-EE group, compared to that in mice from the PD-SC group. The expression of SNAP-25 tended to increase in mice from the PD-EE group, compared to that in mice from the PD-SC group (*n* = 5, each). (**C**) qRT-PCR results showed that the relative mRNA expression of Drd1 was significantly decreased in mice from the PD-EE group, compared to that in mice from the PD-SC group, but the expression of Slc6a3 was significantly increased in mice from the PD-EE group, compared to that in mice from the PD-SC group. The expression of Drd2 between the mice from the PD-SC and PD-EE groups did not differ significantly (*n* = 4, each). (**D**) Western blot results showing the relative protein expression levels of Drd1, Drd2, and Slc6a3 (*n* = 5, each). * *p* < 0.05, ** *p* < 0.01, and *** *p* < 0.001 versus WT control group (WT-CON); one-way ANOVA followed by post-hoc comparison. Data in all panels represent the mean ± SEM.

The qRT-PCR and Western blotting analyses showed the effects of EE on the expression of SNARE proteins, such as SNAP-25, syntaxin1, and VAMP2, in the striatum and NAc of 10-month-old hA53T α-Syn mice. The qRT-PCR results showed that the decreased SNAP-25, syntaxin1, and VAMP2 mRNA expression in the mice from the PD-SC group tended to be restored in the mice from the PD-EE group, compared to the case in the mice from the WT control group (Figure 3A). The Western blot results indicated that the relative protein levels of SNARE proteins were significantly increased in mice from the PD-EE group, compared to those in the mice from the PD control group (Figure 3B).

Next, we investigated the effects of EE on the expression of dopamine receptors and transporters, such as Drd1, Drd2, and Slc6a3, in the striatum and NAc of 10-month-old hA53T α-Syn mice. The RT-qPCR results showed that the relative mRNA expression of Drd1 was significantly decreased in the mice from the PD-EE group, compared to that in the mice from the PD-SC group, but the expression of Slc6a3 was significantly increased in the mice from the PD-EE group, compared to that in the mice from the PD-SC group (Figure 3C). The expression of Drd2 in the mice from the PD-SC and PD-EE groups did not differ significantly. The Western blotting results also showed that the relative protein expression of Drd1 was significantly decreased in mice from the PD-EE group, compared to that in mice from the PD-SC group (Figure 3D). The relative protein expression of Slc6a3 was significantly higher in mice from the PD-EE group than in those from the PD-SC group. The expression of Drd2 in mice from the PD-SC and PD-EE groups did not differ significantly.

### 3.4. EE Reduces Aggregated α-Syn Levels and the Interaction Between α-Syn and VAMP-2 in the NAc of hA53T α-Syn-Overexpressing Transgenic Mice

**Figure 4:**
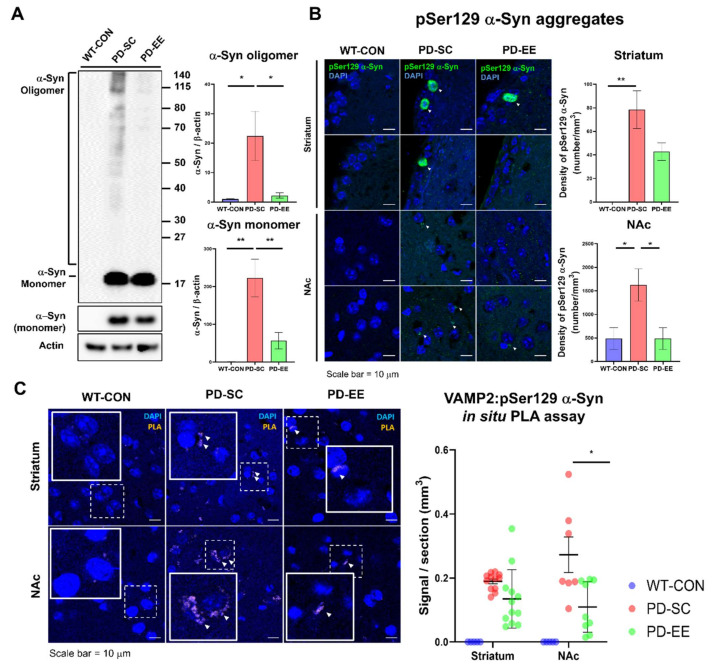
EE reduced the levels of aggregated α-Syn and the interaction between α-Syn and VAMP-2 in the NAc of hA53T α-Syn-overexpressing transgenic mice. (**A**) Western blot results indicated the relative expression levels of the α-Syn monomer and oligomer. The expression of the α-Syn monomer tended to increase but that of aggregated α-Syn decreased in samples from mice in the PD-EE group, compared to those in the samples from mice in the PD-SC group (*n* = 5, each). (**B**) Representative images of pSer129 α-Syn immunohistochemistry in the striatum and NAc of the mice from three groups. The density of pSer129 α-Syn tended to decrease in samples from mice in the PD-EE group compared to that in the samples from mice in the PD-SC group in both regions (scale bar = 10 μm, striatum: WT control group (WT-CON), *n* = 5; PD-SC, *n* = 8; PD-EE, *n* = 6; NAc: WT-CON, *n* = 5; PD-SC, *n* = 6; PD-EE, *n* = 5). (**C**) The proximity of VAMP2 and pSer129 α-Syn in the striatum and NAc, assessed by proximity ligation assay, showed decreased PLA signals in samples from mice in the PD-EE group, relative to that in samples from mice in the PD-SC group (scale bar = 10 μm, striatum: *n* = 4 each; NAc: WT-CON, *n* = 4; PD-SC, *n* = 3; PD-EE, *n* = 3). * *p* < 0.05, ** *p* < 0.01 versus WT-CON; one-way ANOVA followed by post-hoc comparison. Data in all panels represent the mean ± SEM.

Next, we examined whether EE can block α-Syn aggregation because the A53T mutation of α-Syn can increase its tendency to aggregate [45]. The intensity of the α-Syn monomer band (14 kDa) was higher in the samples from the PD-SC and PD-EE groups than in those from the WT control group (Figure 4A). Levels of monomeric and α-Syn aggregates were slightly decreased in samples from the PD-EE group, compared to those in samples from the PD-SC group. Interestingly, for these tendencies, the increased expression of the α-Syn monomer and the decreased expression of the α-Syn oligomer in the samples from the PD-EE group were consistent with those in a previous study involving human α-Syn-expressing transgenic mice [46].

IHC was performed to investigate the effects of EE on the aggregation of α-Syn in the striatum and NAc in 10-month-old mice. The IHC results showed that the pSer129 α-Syn counts tended to decrease in samples from the PD-EE group, compared to those in samples from the PD-SC group (Figure 4B). The total α-Syn in the striatum and nucleus accumbens (NAc) of human A53T α-Syn mice was shown in Figure S1.

pSer129 α-Syn directly binds to the SNARE-protein VAMP2 [10], and α-Syn-overexpressing mice showed inhibited intersynaptic vesicle mobility and trafficking [9]. To confirm that EE affected the interaction between pSer129 α-Syn and VAMP2, an in situ PLA assay was conducted using the striatum and NAc samples of mice from the PD-SC and PD-EE groups. Red puncta indicating the complexes formed between pSer129 α-Syn and VAMP2 were observed. An abundant signal was observed in the NAc of mice from the PD-SC group, but not in the samples of mice from the PD-EE group (Figure 4C). There were no differences between the levels of interaction between pSer129 α-Syn and VAMP2 in the striatum samples from hA53T α-Syn-overexpressing transgenic mice in the PD-SC and PD-EE groups. We confirmed that EE can reduce the interaction between VAMP2 and pSer129 α-Syn in the NAc using an in situ PLA assay.

## 4. Discussion

Motor impairments in patients with PD are primarily due to a 50–70% loss of dopamine neurons in the SNpc [47]. The mouse model used in this study, which expresses hA53T α-Syn under the control of the mouse Prnp promoter, did not show degeneration in the SNpc even at 13–14 months old, which was consistent with the results shown in Figure 2 [48]. Dopaminergic neurons in the striatum of 10-month-old hA53T α-Syn-overexpressing transgenic mice showed only mild degeneration (~20%), compared to the case in the mice from the WT control group. 

The pathogenesis of PD is associated with an increased degree of α-Syn pathology [18]. The manifestation of non-motor symptoms, such as depression and anxiety, is related to neurochemical changes and the brain reward circuit, including the NAc, in PD [19,22]. Anxiety is frequent and can be the starting signal of the disease before the onset of motor defects in PD [49,50]. Dysregulation of dopamine levels in the NAc is related to hyperactivity disorders [51].

Abnormal accumulation of Lewy bodies is a characteristic of PD and is associated with many neurodegenerative diseases. A recent study revealed that a higher level of impulsive compulsive behaviors in PD is associated with the increased expression of α-Syn proteins and dopamine receptors in the NAc of patients with PD [52]. However, the physiological roles of α-Syn in the non-motor symptoms of PD are yet to be fully elucidated. Consistent with the findings of a previous study [53,54], the present study showed that the mice did not show motor deficits in the grip strength test, hanging wire test, or cylinder test, but non-motor symptoms, such as anxiety and hyperactivity, were revealed in the mice, as shown by the open field test results in Figure 1F,G.

Numerous studies have shown that α-Syn, in the control center of neurotransmitter delivery, regulates the formation of the SNARE complex and the size of the synaptic vesicle pool [9,10,55,56]. Recent studies have shown that pSer129 α-Syn proteins can attach to SNARE proteins and interrupt their functions [10,57]. In particular, the A53T mutation of α-Syn is a point mutation that causes familial PD. Many studies have reported progressive motor dysfunction [1,2] and non-motor dysfunction [3,4,5] in mice with PD that overexpress hA53T α-Syn. The dysfunction of axonal transportation by synaptophysin-positive vesicles has also been shown in a hA53T transgenic α-Syn model [6].

EE, which involves a large cage containing physical, cognitive, and social stimuli, can be used as a therapy for PD (Figure 1B). Several studies have investigated the effects of EE on PD and found that EE alleviates behavioral deficits in a mouse model of PD with MPTP administration [7,8]. In our previous study, EE also upregulated synaptic vesicle-associated proteins in the striatum of normal Institute of Cancer Research (ICR) mice [9]. Therefore, in this study, we aimed to identify the therapeutic effects of EE on familial PD in terms of synaptic transmission in the striatum and NAc.

Consistent with the results of a previous study [7,8,9] exposure to EE improved the expression of SNAP-25, syntaxin1, and VAMP2 and decreased the pathological α-Syn levels simultaneously (Figure 3), unlike the case in hA53T α-Syn-overexpressing mice at 10 months of age. We confirmed that EE can suppress the interaction between VAMP2 and pSer129 α-Syn via an in situ PLA assay (Figure 4C).

The upregulation of Drd1 is a compensatory response associated with deficient dopamine signaling [58,59]. Kurz et al. reported increased levels of striatal dopamine in both young (eight months old) and old (18 months old) hA53T α-Syn-overexpressing mice and elevated levels of striatal Drd1 without any indication of neurodegeneration [58]. Furthermore, as a compensatory effect, the expression of Drd1 could be increased to enable the reception of more synaptic signals.

The expression of Slc6a3, a dopamine transporter protein, decreased in mice from the PD-SC group but was restored by EE. Erica et al. confirmed the decreased expression of this dopamine transporter in the NAc and striatum and the reduction of dopamine uptake in 10-month-old hA53T α-Syn transgenic mice [15]. Since α-Syn reduces dopamine uptake by decreasing the activity of this dopamine transporters, the overexpression of α-Syn may further decrease the levels of this dopamine transporters in hA53T α-Syn-overexpressing transgenic mice. 

Previous studies have investigated the effect of EE on normal mice. Long-term exposure to EE altered synaptic activity-regulating genes underlying functional improvement in the adult brain [60]. Moreover, EE induced synaptic plasticity through the internalization of striatal dopamine transporter [61], and upregulated the expression of synaptic vesicle-associated proteins [62]. In this study, we focused on the effect of EE on PD in terms of synaptic vesicle trafficking, based on the previous studies. 

This study had limitations, in that we only included the WT-CON group as a baseline for molecular and histological assessments. Since the WT-EE group can be an indicator for the improvement of synaptic transmission, further studies including the WT-EE group should be conducted to investigate the mechanism underlying the action of EE.

**Figure 5:**
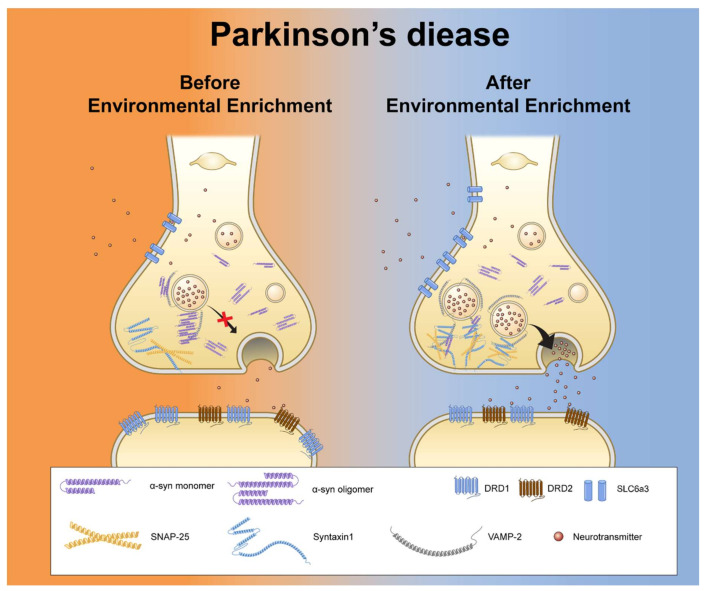
Synopsis of the amelioration of aberrant synaptic signaling in hA53T α-Syn-overexpressing transgenic mice by EE. This schematic illustration summarizes the progressive pathology of dopaminergic nerve terminals in the striatum and NAc in PD. Initially, the functions of SNAP-25, syntaxin1, and VAMP2 help synaptic vesicles to dock and fuse to permit exocytosis. These functions are interrupted by aggregated α-Syn proteins; therefore, dopamine release decreased in the mice from the PD-SC group. As a compensatory effect, the expression of Drd1 increased, enabling the reception of more synaptic signals. EE simultaneously reduced the levels of aggregated α-Syn and enhanced the expression of SNARE proteins, such as syntaxin1 and VAMP2. SNARE proteins may recover their functions. Finally, synaptic vesicles were re-circulated and normalized; thus, cytosolic dopamine levels may be normalized in the NAc. Since the activities of dopamine transporters and/or dopamine receptors were normalized, pre-motor symptoms were ameliorated, and the progression of the disease could be delayed.

In this study, we identified progressively increasing striatal dopamine levels as an early effect of hA53T α-Syn overexpression prior to neurodegeneration (Figure 5). The results of this study suggest that EE exerts therapeutic effects on the early symptoms of PD, including hyperactivity and anxiety, as it is mainly responsible for the expression of synaptic proteins, dopamine transporters, and dopamine receptors.

## 5. Conclusions

In this study, we confirmed the potential therapeutic effects of EE on the non-motor symptoms of PD. Non-motor symptoms, such as hyperactivity and anxiety, were ameliorated by EE through the upregulation of synaptic vesicle proteins, such as SNAP-25, syntaxin1, and VAMP2, and by suppressing the accumulation of aggregated α-Syn in the NAc and striatum. The expression of Drd1 was upregulated in mice from the PD-SC group, compared to that in mice from the WT control group. This might be a compensatory response to dopamine deficiency. However, the expression of Slc6a3 was downregulated in mice from the PD-SC group, which was rescued by EE. Our results highlight the importance of the therapeutic effects of EE on PD, which is associated with the modulation of synaptic vesicle proteins, dopamine receptors, and dopamine transporters. 

## Data Availability

Data is contained within the article or supplementary material. The data presented in this study are available.

## Supplementary Materials

The following are available online at https://www.mdpi.com/2073-4425/12/3/392/s1, Figure S1: The total α-synuclein (α-Syn) in the striatum and nucleus accumbens (NAc) of human A53T α-Syn mice.

## References

[1] Spillantini M.G., Crowther R.A., Jakes R., Cairns N.J., Lantos P.L., Goedert M. (1998). Filamentous α-synuclein inclusions link multiple system atrophy with Parkinson’s disease and dementia with Lewy bodies. Neurosci. Lett..

[2] Spillantini M.G., Crowther R.A., Jakes R., Hasegawa M., Goedert M. (1998). α-Synuclein in filamentous inclusions of Lewy bodies from Parkinson’s disease and dementia with Lewy bodies. Proc. Natl. Acad. Sci. USA.

[3] Spillantini M.G., Schmidt M.L., Lee V.M.-Y., Trojanowski J.Q., Jakes R., Goedert M. (1997). α-Synuclein in Lewy bodies. Nature.

[4] Murphy D.D., Rueter S.M., Trojanowski J.Q., Lee V.M.-Y. (2000). Synucleins are developmentally expressed, and α-synuclein regulates the size of the presynaptic vesicular pool in primary hippocampal neurons. J. Neurosci..

[5] Yavich L., Tanila H., Vepsäläinen S., Jäkälä P. (2004). Role of α-synuclein in presynaptic dopamine recruitment. J. Neurosci..

[6] Cabin D.E., Shimazu K., Murphy D., Cole N.B., Gottschalk W., McIlwain K.L., Orrison B., Chen A., Ellis C.E., Paylor R. (2002). Synaptic vesicle depletion correlates with attenuated synaptic responses to prolonged repetitive stimulation in mice lacking α-synuclein. J. Neurosci..

[7] Choi B.-K., Choi M.-G., Kim J.-Y., Yang Y., Lai Y., Kweon D.-H., Lee N.K., Shin Y.-K. (2013). Large α-synuclein oligomers inhibit neuronal SNARE-mediated vesicle docking. Proc. Natl. Acad. Sci. USA.

[8] Nemani V.M., Lu W., Berge V., Nakamura K., Onoa B., Lee M.K., Chaudhry F.A., Nicoll R.A., Edwards R.H. (2010). Increased expression of α-synuclein reduces neurotransmitter release by inhibiting synaptic vesicle reclustering after endocytosis. Neuron.

[9] Scott D., Roy S. (2012). α-Synuclein inhibits intersynaptic vesicle mobility and maintains recycling-pool homeostasis. J. Neurosci..

[10] Burré J., Sharma M., Tsetsenis T., Buchman V., Etherton M.R., Südhof T.C. (2010). α-Synuclein promotes SNARE-complex assembly in vivo and in vitro. Science.

[11] Lou X., Kim J., Hawk B.J., Shin Y.-K. (2017). α-Synuclein may cross-bridge v-SNARE and acidic phospholipids to facilitate SNARE-dependent vesicle docking. Biochem. J..

[12] Giasson B.I., Duda J.E., Quinn S.M., Zhang B., Trojanowski J.Q., Lee V.M.-Y. (2002). Neuronal α-synucleinopathy with severe movement disorder in mice expressing A53T human α-synuclein. Neuron.

[13] Lee M.K., Stirling W., Xu Y., Xu X., Qui D., Mandir A.S., Dawson T.M., Copeland N.G., Jenkins N.A., Price D.L. (2002). Human α-synuclein-harboring familial Parkinson’s disease-linked Ala-53→ Thr mutation causes neurodegenerative disease with α-synuclein aggregation in transgenic mice. Proc. Natl. Acad. Sci. USA.

[14] Farrell K.F., Krishnamachari S., Villanueva E., Lou H., Alerte T.N., Peet E., Drolet R.E., Perez R.G. (2014). Non-motor parkinsonian pathology in aging A53T α-Synuclein mice is associated with progressive synucleinopathy and altered enzymatic function. J. Neurochem..

[15] Unger E.L., Eve D.J., Perez X.A., Reichenbach D.K., Xu Y., Lee M.K., Andrews A.M. (2006). Locomotor hyperactivity and alterations in dopamine neurotransmission are associated with overexpression of A53T mutant human α-synuclein in mice. Neurobiol. Dis..

[16] Wang W., Song N., Jia F., Tang T., Bao W., Zuo C., Xie J., Jiang H. (2018). Genomic DNA levels of mutant alpha-synuclein correlate with non-motor symptoms in an A53T Parkinson’s disease mouse model. Neurochem. Int..

[17] Koch J., Bitow F., Haack J., d’Hedouville Z., Zhang J., Tönges L., Michel U., Oliveira L., Jovin T., Liman J. (2015). Alpha-Synuclein affects neurite morphology, autophagy, vesicle transport and axonal degeneration in CNS neurons. Cell Death Dis..

[18] Riley B.E., Gardai S.J., Emig-Agius D., Bessarabova M., Ivliev A.E., Schüle B., Alexander J., Wallace W., Halliday G.M., Langston J.W. (2014). Systems-based analyses of brain regions functionally impacted in Parkinson’s disease reveals underlying causal mechanisms. PLoS ONE.

[19] Carriere N., Besson P., Dujardin K., Duhamel A., Defebvre L., Delmaire C., Devos D. (2014). Apathy in Parkinson’s disease is associated with nucleus accumbens atrophy: A magnetic resonance imaging shape analysis. Mov. Disord..

[20] Juárez Olguín H., Calderon Guzman D., Hernandez Garcia E., Barragán Mejía G. (2016). The role of dopamine and its dysfunction as a consequence of oxidative stress. Oxid. Med. Cell. Longevity.

[21] Russo S.J., Nestler E.J. (2013). The brain reward circuitry in mood disorders. Nat. Rev. Neurosci..

[22] Kano O., Ikeda K., Cridebring D., Takazawa T., Yoshii Y., Iwasaki Y. (2011). Neurobiology of depression and anxiety in Parkinson’s disease. Parkinson’s Dis..

[23] Iarkov A., Barreto G.E., Grizzell J.A., Echeverria V. (2020). Strategies for the treatment of Parkinson’s disease: Beyond dopamine. Front. Aging Neurosci..

[24] Salgado S., Kaplitt M.G. (2015). The nucleus accumbens: A comprehensive review. Stereotact. Funct. Neurosurg..

[25] Williams N., Short B., Williams E., Jeffery A., Kerns S., Sahlem G., Hanlon C., Revuelta G., Takacs I., George M. (2014). Deep Brain Stimulation of the Nucleus Accumbens Has Positive Effects on Parkinson’s Disease-Related Apathy (P7.050). Neurology.

[26] Rosenzweig M.R., Bennett E.L., Hebert M., Morimoto H. (1978). Social grouping cannot account for cerebral effects of enriched environments. Brain Res..

[27] Janssen H., Ada L., Bernhardt J., McElduff P., Pollack M., Nilsson M., Spratt N.J. (2014). An enriched environment increases activity in stroke patients undergoing rehabilitation in a mixed rehabilitation unit: A pilot non-randomized controlled trial. Disabil. Rehabil..

[28] White J.H., Alborough K., Janssen H., Spratt N., Jordan L., Pollack M. (2014). Exploring staff experience of an “enriched environment” within stroke rehabilitation: A qualitative sub-study. Disabil. Rehabil..

[29] Chen H., Zhang S., Schwarzschild M., Hernan M., Ascherio A. (2005). Physical activity and the risk of Parkinson disease. Neurology.

[30] Xu Q., Park Y., Huang X., Hollenbeck A., Blair A., Schatzkin A., Chen H. (2010). Physical activities and future risk of Parkinson disease. Neurology.

[31] Berchtold N.C., Castello N., Cotman C.W. (2010). Exercise and time-dependent benefits to learning and memory. Neuroscience.

[32] Cotman C.W., Berchtold N.C. (2007). Physical activity and the maintenance of cognition: Learning from animal models. Alzheimers Dement.

[33] Petzinger G.M., Fisher B.E., Van Leeuwen J.E., Vukovic M., Akopian G., Meshul C.K., Holschneider D.P., Nacca A., Walsh J.P., Jakowec M.W. (2010). Enhancing neuroplasticity in the basal ganglia: The role of exercise in Parkinson’s disease. Mov. Disord..

[34] Faherty C.J., Shepherd K.R., Herasimtschuk A., Smeyne R.J. (2005). Environmental enrichment in adulthood eliminates neuronal death in experimental Parkinsonism. Mol. Brain. Res..

[35] Goldberg N., Haack A., Meshul C. (2011). Enriched environment promotes similar neuronal and behavioral recovery in a young and aged mouse model of Parkinson’s disease. Neuroscience.

[36] Fan L.-W., Lin S., Pang Y., Lei M., Zhang F., Rhodes P.G., Cai Z. (2005). Hypoxia-ischemia induced neurological dysfunction and brain injury in the neonatal rat. Behav. Brain Res..

[37] Im S., Yu J., Park E., Lee J., Kim H., Park K.I., Kim G., Park C., Cho S.-R. (2010). Induction of striatal neurogenesis enhances functional recovery in an adult animal model of neonatal hypoxic-ischemic brain injury. Neuroscience.

[38] Carola V., D’Olimpio F., Brunamonti E., Mangia F., Renzi P. (2002). Evaluation of the elevated plus-maze and open-field tests for the assessment of anxiety-related behaviour in inbred mice. Behav. Brain Res..

[39] Prut L., Belzung C. (2003). The open field as a paradigm to measure the effects of drugs on anxiety-like behaviors: A review. Eur. J. Pharmacol..

[40] Park H., Chang K.-A. (2020). Therapeutic Potential of Repeated Intravenous Transplantation of Human Adipose-Derived Stem Cells in Subchronic MPTP-Induced Parkinson’s Disease Mouse Model. Int. J. Mol. Sci..

[41] Che Y., Hou L., Sun F., Zhang C., Liu X., Piao F., Zhang D., Li H., Wang Q. (2018). Taurine protects dopaminergic neurons in a mouse Parkinson’s disease model through inhibition of microglial M1 polarization. Cell Death Dis..

[42] Denenberg V.H. (1969). Open-field behavior in the rat: What does it mean?. Ann. N. Y. Acad. Sci..

[43] Walsh R.N., Cummins R.A. (1976). The open-field test: A critical review. Psychol. Bull..

[44] Valvassori S.S., Varela R.B., Quevedo J. (2017). Animal models of mood disorders: Focus on bipolar disorder and depression. Animal Models for the Study of Human Disease.

[45] Ostrerova-Golts N., Petrucelli L., Hardy J., Lee J.M., Farer M., Wolozin B. (2000). The A53T α-synuclein mutation increases iron-dependent aggregation and toxicity. J. Neurosci..

[46] Zhou W., Barkow J.C., Freed C.R. (2017). Running wheel exercise reduces α-synuclein aggregation and improves motor and cognitive function in a transgenic mouse model of Parkinson’s disease. PLoS ONE.

[47] Fearnley J.M., Lees A.J. (1991). Ageing and Parkinson’s disease: Substantia nigra regional selectivity. Brain.

[48] Daher J.P.L., Pletnikova O., Biskup S., Musso A., Gellhaar S., Galter D., Troncoso J.C., Lee M.K., Dawson T.M., Dawson V.L. (2012). Neurodegenerative phenotypes in an A53T α-synuclein transgenic mouse model are independent of LRRK2. Hum. Mol. Genet..

[49] Pellicano C., Benincasa D., Pisani V., Buttarelli F.R., Giovannelli M., Pontieri F.E. (2007). Prodromal non-motor symptoms of Parkinson’s disease. Neuropsychiatr. Dis. Treat..

[50] Shiba M., Bower J.H., Maraganore D.M., McDonnell S.K., Peterson B.J., Ahlskog J.E., Schaid D.J., Rocca W.A. (2000). Anxiety disorders and depressive disorders preceding Parkinson’s disease: A case-control study. Mov. Disord..

[51] Dagher A., Robbins T.W. (2009). Personality, addiction, dopamine: Insights from Parkinson’s disease. Neuron.

[52] Barbosa P., Hapuarachchi B., Djamshidian A., Strand K., Lees A.J., de Silva R., Holton J.L., Warner T.T. (2019). Lower nucleus accumbens α-synuclein load and D3 receptor levels in Parkinson’s disease with impulsive compulsive behaviours. Brain.

[53] Guerreiro P.S., Coelho J.E., Sousa-Lima I., Macedo P., Lopes L.V., Outeiro T.F., Pais T.F. (2017). Mutant A53T α-Synuclein Improves Rotarod Performance Before Motor Deficits and Affects Metabolic Pathways. Neuromol. Med..

[54] Peters S.T., Fahrenkopf A., Choquette J.M., Vermilyea S.C., Lee M.K., Vossel K. (2020). Ablating tau reduces hyperexcitability and moderates electroencephalographic slowing in transgenic mice expressing A53T human α-synuclein. Front. Neurol..

[55] Darios F., Ruiperez V., Lopez I., Villanueva J., Gutierrez L.M., Davletov B. (2010). α-Synuclein sequesters arachidonic acid to modulate SNARE-mediated exocytosis. EMBO Rep..

[56] Chandra S., Gallardo G., Fernández-Chacón R., Schlüter O.M., Südhof T.C. (2005). Alpha-synuclein cooperates with CSPalpha in preventing neurodegeneration. Cell.

[57] Garcia-Reitböck P., Anichtchik O., Bellucci A., Iovino M., Ballini C., Fineberg E., Ghetti B., Della Corte L., Spano P., Tofaris G.K. (2010). SNARE protein redistribution and synaptic failure in a transgenic mouse model of Parkinson’s disease. Brain.

[58] Kurz A., Double K.L., Lastres-Becker I., Tozzi A., Tantucci M., Bockhart V., Bonin M., García-Arencibia M., Nuber S., Schlaudraff F. (2010). A53T-alpha-synuclein overexpression impairs dopamine signaling and striatal synaptic plasticity in old mice. PLoS ONE.

[59] Seeman P., Niznik H.B. (1990). Dopamine receptors and transporters in Parkinson’s disease and schizophrenia. FASEB J..

[60] Lee M.-Y., Yu J.H., Kim J.Y., Seo J.H., Park E.S., Kim C.H., Kim H., Cho S.-R. (2013). Alteration of synaptic activity–regulating genes underlying functional improvement by long-term exposure to an enriched environment in the adult brain. Neurorehabilit. Neural Repair.

[61] Kim M.-S., Yu J.H., Kim C.H., Choi J.Y., Seo J.H., Lee M.-Y., Yi C.H., Choi T.H., Ryu Y.H., Lee J.E. (2016). Environmental enrichment enhances synaptic plasticity by internalization of striatal dopamine transporters. J. Cereb. Blood Flow Metab..

[62] Song S.-Y., Chae M., Yu J.H., Lee M.Y., Pyo S., Shin Y.-K., Baek A., Park J.-W., Park E.S., Choi J.Y. (2018). Environmental enrichment upregulates striatal synaptic vesicle-associated proteins and improves motor function. Front. Neurol..

