# Supplementary material for: Reduced Interaction of Aggregated α-Synuclein and VAMP2 by Environmental Enrichment Alleviates Hyperactivity and Anxiety in a Model of Parkinson’s Disease
Source: Genes (Basel). 2021 Mar 10;12(3):392. doi: 10.3390/genes12030392 (PMC7998569; doi:10.3390/genes12030392)
Supplement: Supplementary file 1 [file genes-12-00392-s001.pdf]

Supplementary Figure

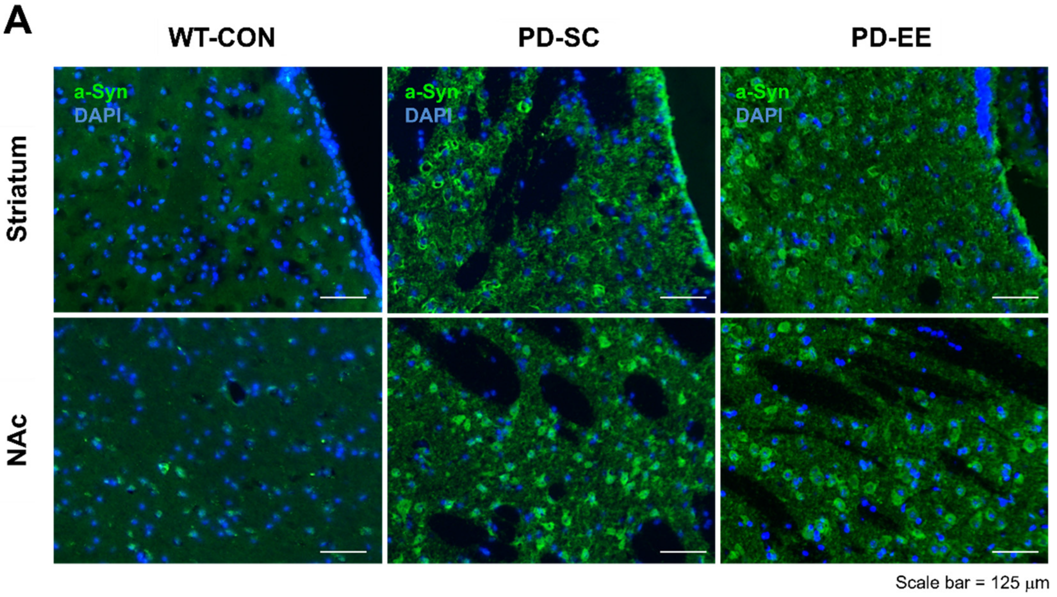

Figure S1. The total α-synuclein (α-Syn) in the striatum and nucleus accumbens (NAc) of human A53T α-Syn mice. The PD group showed a significant increase in the expression of α-Syn compared to the WT control group. (**A**) Scale bar = 500 μm. (**B**) Scale bar = 125 μm. (**C**) Scale bar = 10 μm.

Supplementary Materials and Methods

1. Immunohistochemistry (IHC)

The animals were euthanized and perfused with 4% paraformaldehyde (PFA) in 0.1 M phosphate buffer, pH 7.4. Their brains were removed and post-fixed for 1 h, followed by cryoprotection in 30% sucrose in TBS containing 0.02% sodium azide. The harvested brain tissues were cryo-sectioned at a thickness of 16 μm along the sagittal or coronal plane, and IHC analysis was performed on four sections. For immunofluorescence double labeling, the sections were stained with the following antibodies: antibodies against α-Syn (1:200, Abcam, ab138501, Cambridge, UK) and secondary antibody, Alexa Fluor® 488 goat anti-rabbit (1:400, Invitrogen, A110088, Carlsbad, CA, USA). The sections were mounted on glass slides with fluorescent mounting medium containing 4',6-diamidino-2-phenylindole (Vector, H-1200; Vector, Burlingame, CA, USA). The stained sections were examined using a fluorescence microscope (Axio Imager M2, Zeiss, Gottingen, Germany) and a confocal microscope (LSM700, Zeiss, Gottingen, Germany).
